# Stepwise Structural Relaxation in Battery Active Materials

**DOI:** 10.1021/acsmaterialslett.4c02058

**Published:** 2024-12-23

**Authors:** Amalie Skurtveit, Erlend Tiberg North, Heesoo Park, Dmitry Chernyshov, David S. Wragg, Alexey Y. Koposov

**Affiliations:** † Centre for Materials Science and Nanotechnology, Department of Chemistry, University of Oslo, PO Box 1033, Blindern 0315 Norway; ‡ Swiss-Norwegian Beamlines, European Synchrotron Facility, 71 Avenue des Martyrs, 38000 Grenoble, France; § Department of Battery Technology, Institute for Energy Technology, Instituttveien 18, 2007 Kjeller, Norway

## Abstract

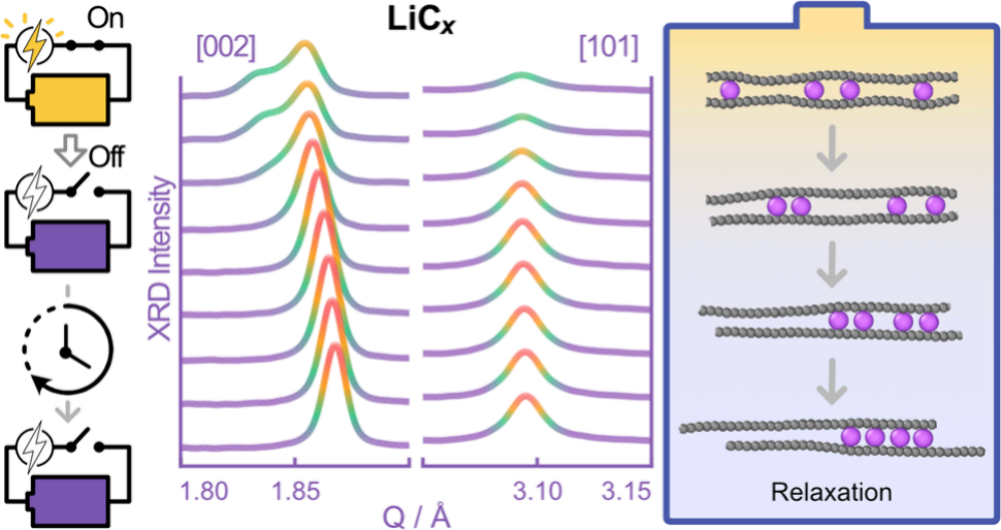

Whenever the cycling of Li-ion batteries is stopped, the electrode materials undergo a relaxation process, but the structural changes that occur during relaxation are not well-understood. We have used operando synchrotron X-ray diffraction with a time resolution of 1.24 s to observe the structural changes that occur when the lithiation of graphite and LiFePO_4_ electrodes are interrupted. Assessing the kinetics of the relaxation processes coupled with molecular dynamics simulations allows us to identify three relaxation stages in graphite. The atomistic origin for the relaxation process within the partially lithiated graphite structure is driven by the reorganization of Li ions into Li clusters. Relaxation in LiFePO_4_ electrodes is considerably slower than for graphite, but the observed structural changes is also attributed to reorganization of Li ions. These insights highlight the nature of the structural changes that occur during relaxation and the importance of using operando structural studies to avoid misleading conclusions about the reaction mechanisms in battery materials.

The development and optimization of materials for rechargeable batteries depend on detailed characterization, which links the structural evolution of a material with its electrochemical behavior. The majority of characterization techniques used for the analysis of battery materials today rely on the temporary or permanent interruption of electrochemical cycling.^1−5^ These interruptions lead to relaxation processes in the active materials. Despite extensive studies of (dis)charge mechanisms in multiple chemistries, the structural origin of relaxation is not well understood.^6−14^ However, active materials often undergo structural transformations through highly reactive and metastable phases during cycling; therefore, the state of the material is likely to change from the moment of electrochemical interruption to the moment of actual analyses.^7,15−17^ These changes could affect not only the results obtained from post-mortem/ex situ structural analyses but also the further cyclability of the materials and electrochemical results.

Although many studies have reported relaxation of battery active materials, none to date have considered their structural origins and mechanisms. Several publications demonstrated relaxation processes in materials when subjected to high cycling rates aiming to capture the formation of metastable intermediate phases during electrochemical cycling or open-circuit-voltage to observe reorganization of Li ions in graphite/silicon electrodes.^9,12^ Redistribution of Li ions between neighboring particles due to nonuniform lithiation has also been proposed through modeling experiments for LiFePO_4_ (LFP) and Si-based electrodes.^18−20^ In addition, time-resolved ex-situ X-ray diffraction (XRD) studies have shown that materials extracted from working cells may change structure from 30 min to 51 h after extraction.^21,22^ Furthermore, the interactions of alkali-metal ions in metal-ion batteries might proceed nonuniformly and may lead to redistribution of ions in the host structure, as has been shown for graphite and LFP in Li-ion batteries (LIBs).^22−24^

Electrochemical techniques, such as galvanostatic intermittent titration technique (GITT), utilize relaxation processes in active materials to analyze ionic diffusion in batteries.^13,14,25^ GITT has been widely applied for characterization of materials and estimation of diffusion coefficients for multiple battery chemistries.^26−28^ However, since the structural mechanisms of relaxation are not understood, misleading parametrization can lead to discrepancies in the reported diffusion coefficients obtained. As a result, the reported Li-diffusion coefficient in graphite estimated from GITT experiments varies between 10^–13^ cm^2^ s^–1^ and 10^–11^ cm^2^ s^–1^,^14,28^ while that of LFP has been reported to be between 10^–16^ cm^2^ s^–1^ and 10^–8^ cm^2^ s^–1^.^29^ It should be noted that diffusion coefficients are affected by the concentration of Li ions. The lack of knowledge concerning the structural changes during relaxation impedes the further development of battery materials and cycling protocols for battery systems.

To understand and reveal the structural evolution of active battery materials during relaxation, we selected two common battery materials, graphite and LFP, and studied them with operando XRD after the interruption of electrochemical cycling. Operando XRD conducted at the European Synchrotron Radiation Facility (ESRF) with 1.24 s time resolution allowed us to follow the evolution of the structure directly and capture short-lived intermediate states.^3,9^ The selected experimental conditions resemble those encountered before post-mortem and in-situ analysis of battery materials and/or during the relaxation step of GITT. Molecular dynamics simulations allowed us to interpret these observations based on the reorganization of Li ions within the structure of graphite, and the kinetics of graphite relaxation were studied using Kolmogorov–Johnson–Mehl–Avrami (KJMA) theory.

The evolution of the synchrotron operando XRD of the graphite-based electrode extracted from a commercial LG JP3 cell with a particle size of ∼10 μm cycled galvanostatically at C/6 is shown in Figure 1. The (002) reflection was continuously monitored with a constant time resolution of 1.24 s, as a function of (de)lithiation; such time resolution for operando XRD measurements is achievable only at synchrotron facilities. Shifts in the position of the (002) reflection are attributed to changes in the spacing of the graphene layers (gallery height) and used as a structural marker to describe the operating mechanism of graphite in LIBs and the degree of lithiation, as elucidated through *operando* XRD and neutron diffraction.^30−35^ The (101) reflection is attributed to a plane of atoms affected by both the gallery height and the stacking sequence of the graphene sheets (Figure 1f). For consistency, LiC_*x*_ reflections are named based on the Miller indices of graphite throughout this Letter.

**Figure 1:**
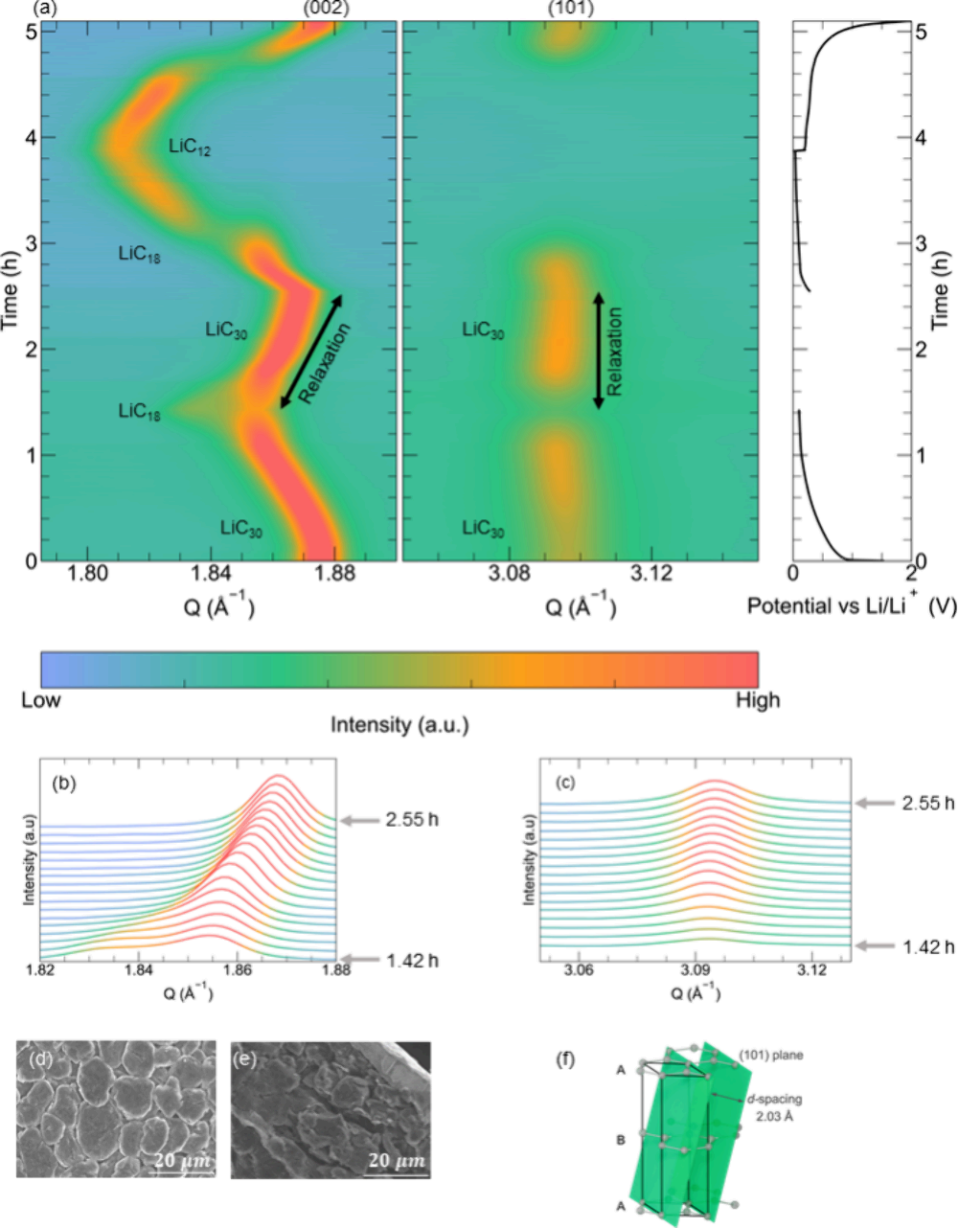
Operando XRD Characterization of graphite during relaxation. (a) Evolution of the (002) and (101) reflections of graphite as a function of (de)lithiation and state of relaxation. Selected scans for tracing diffraction patterns during relaxation (∼1.42–2.55 h after the start of measurement) of (b) the (002) reflection and (c) the (101) reflection. SEM micrographs of the pristine electrodes: (d) top-view and (e) cross-section. (f) Illustration of the (101) plane in graphite.

Immediately after the start of lithiation, the (002) reflection shifts to lower *Q*-values, illustrating the beginning of the Li-intercalation process and the formation of a LiC_30_-type phase. At ∼0.1 V vs Li/Li^+^ (∼1.42 h after the start of the measurement), the appearance of a new (002) reflection at 1.835 Å^–1^ and simultaneous loss of intensity in the initial peak at 1.853 Å^–1^ signal the start of the staging phase transition from LiC_30_ to LiC_18_. At the same time, we observe intensification of the (101) reflection and a small shift to lower *Q* during the lithiation of graphite. When the phase transition from LiC_30_ to LiC_18_ begins, this reflection shifts from 3.09 Å^–1^ to ∼3.16 Å^–1^ and becomes very broad (Figure 1 and Figure S1).

At the onset of the LiC_30_ to LiC_18_ phase transition (∼1.4 h), the potentiostat was physically disconnected from the cell while the diffraction acquisition continued. Within minutes of disconnection, the developing LiC_18_ phase entirely disappeared (i.e., reverse phase transition), and the (002) reflection corresponding to the LiC_30_ phase started to drift back to higher *Q*-values (Figure 1b). After ∼12 min of relaxation, the (101) reflection gradually reappears at 3.09 Å^–1^ (Figure 1c).

The drift of the (002) and (101) reflections to higher *Q*-values continued for ∼1 h before the peak positions stabilized at ∼1.87 Å^–1^ (002) and ∼3.10 Å^–1^ (101). The electrical disconnection means that no additional Li-ions should be delivered to the graphite electrode and, thus, should freeze the lithiation process. However, the observed drift of the reflections to higher *Q*-values indicates that the structure of lithiated graphite continues to evolve. The electrochemical behavior of the electrode was not disrupted by the interruption and structural relaxation as the operando measurement was resumed at ∼C/2 cycling rate after stabilization and continued as normal (∼2.55 h and onward in Figure 1). Operando XRD data on a reference cell built in the same way and cycled at C/20 for the first (de)lithiation (Figure S2) show that the relaxed cell was behaving as expected.

To illustrate that the observed structural relaxation was not material-specific, we analyzed the behavior of a commercial LFP-based electrode (LFP particle size ∼0.5 μm) using the same experimental methodology. LFP is a cathode material whose cycling mechanism has been the subject of multiple studies.^36−39^ Considering the difference in the electrochemical mechanisms of LFP and graphite, we interrupted the lithiation of FePO_4_ (FP) after ∼1 h, when reflections corresponding to LFP started to appear. The evolution of the (200) reflections for both phases is shown in Figure 2a. Compared with graphite, the changes in the XRD patterns were less pronounced, but the detailed analysis of peak intensities corresponding to LFP and FP phases demonstrated clear evidence of the relaxation (Figure 2b). The structural relaxation of the LFP/FP-based electrode was significantly slower than that in graphite. The stabilization of the chemical changes—and, therefore, the stabilization of the XRD pattern—took ∼8 h. As for the relaxation of graphite, these changes can be interpreted as a rearrangement of Li ions within the LFP/FP electrode as the electrochemically driven flow of Li ions has been interrupted.

**Figure 2:**
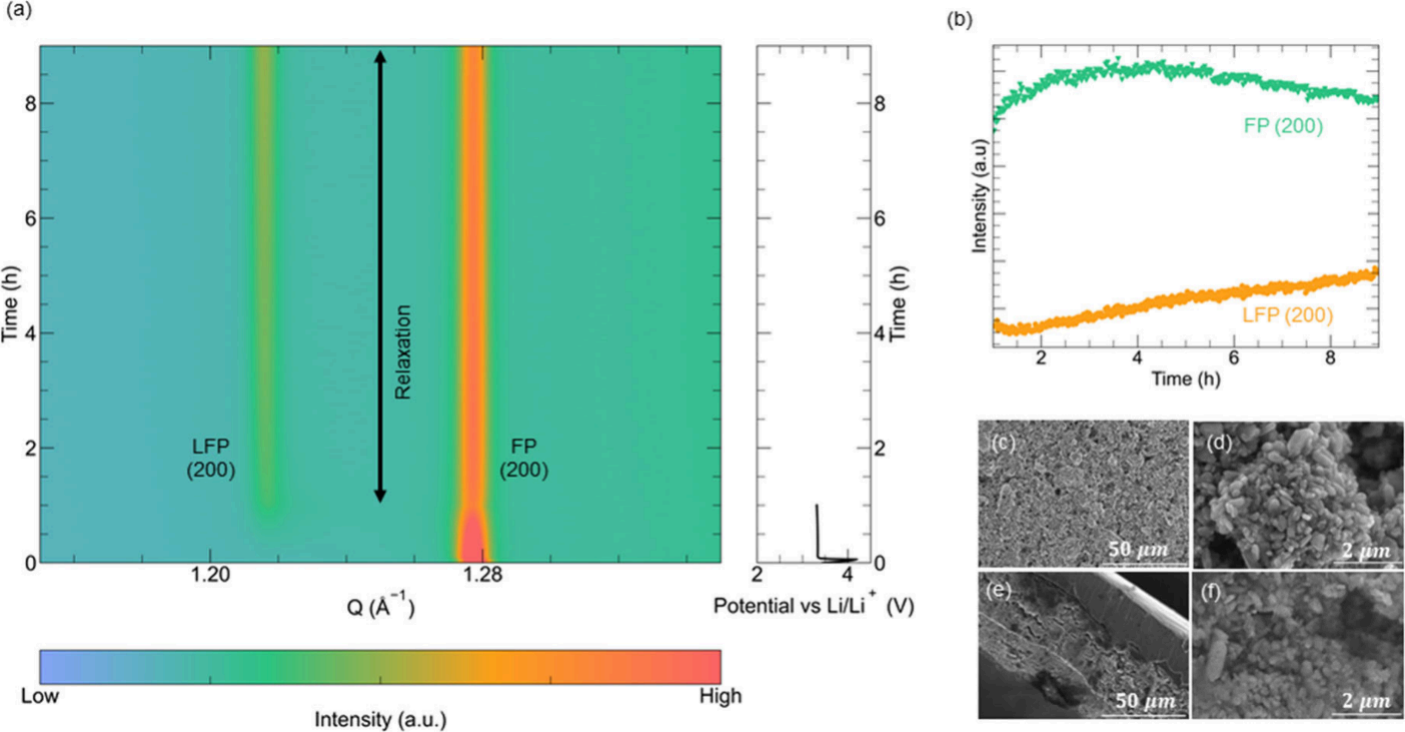
Operando XRD characterization of LiFePO_4_ (LFP) during relaxation. (a) Evolution of the diffraction pattern of FePO_4_ (FP)/LFP as a function of charge and relaxation. (b) Intensity changes during the relaxation of the (200) reflections of LFP/FP as a function of the total time of measurement. SEM micrographs of the pristine electrode (c, d) surface and (e, f) cross-section.

The operando observation of the relaxation processes in both graphite and LFP demonstrates that the nonequilibrium phase formed during Li (de)insertion relaxes toward the most thermodynamically favorable phase. We have intentionally selected both a cathode and an anode material to demonstrate that the relaxation processes are not confined only to a given material or electrode type. We expect that similar effects will occur in other cathode/anode materials in multiple battery chemistries. However, the nature and kinetics of such relaxation processes might be specific to the studied material. Furthermore, the direction and rate of the relaxation process will be determined by the point of interruption of the electrochemistry, material system, and whether the system has already reached a thermodynamically stable phase during the ongoing (dis)charge process. In such a case, relaxation might not be observed at all. To further demonstrate relaxation processes, we have interrupted the electrochemistry another set of cells containing the graphite-based electrodes at other nonequilibrium states, specifically at 0.012 V (corresponding to LiC_18_, Figure S3), and 0.078 V vs Li/Li^+^ (corresponding to pure LiC_30_, Figure S4).

While considering the evolution of the reflections in the operando results above, it is important to note that the XRD patterns represent an average over many local structures, including metastable states within the sample, and that the (de)lithiation process might not always progress by the most thermodynamically favorable intermediates (especially at higher rates of cycling). We attribute the changes in the XRD patterns for both materials to the relaxation of the partially lithiated phases toward their most thermodynamically stable states at that degree of lithiation, by reorganization of the Li ions. There is a strong indication that rearrangement of Li ions within the active materials is preferred based on the changes in the diffraction data (restructuring as a function of relaxation) along with the reversal of the phase changes at the current time scale.^6,21,32^ The alternative of extraction of Li ions from the materials into the electrolyte and SEI is highly unlikely in the observed time scale.^40,41^

To further rationalize the observed relaxation processes in graphite and create models describing the possible structural rearrangements, we conducted replica-exchange molecular dynamics (RE-MD) simulations. RE-MD accelerates the rearrangements of Li ions in the simulation, allowing us to explore representative structures at a given temperature (302.22 K) and degree of lithiation.^42^ For this purpose, we used a composition of LiC_24_, representing the LiC_30_ phase with some additional Li ions–a scenario observed in our experiments (Figure 1). By sampling 64 snapshot trajectories of the LiC_24_ phase, we examined four states denoted LiC_24_-A, LiC_24_-B, LiC_24_-C, and LiC_24_-D. LiC_24_-A has a diffuse Li arrangement with Li ions in all galleries, as would be expected during kinetically limited fast charging. In LiC_24_-B, LiC_24_-C, and LiC_24_-D, we used starting points with empty galleries (similar to stage 2 in the Rüdorff–Hofmann model).^43^ LiC_24_-B and LiC_24_-C contain small Li clusters with a more uniform distribution of Li ions in LiC_24_-C. The last structure, LiC_24_-D, contains Li islands (where an island is referred to large, dense Li clusters).^44^ The simulation allowed us to calculate an XRD pattern corresponding to each lithiated structure. Changes in the (002) reflections, as a function of Li clustering, are shown in Figure 3.

**Figure 3:**
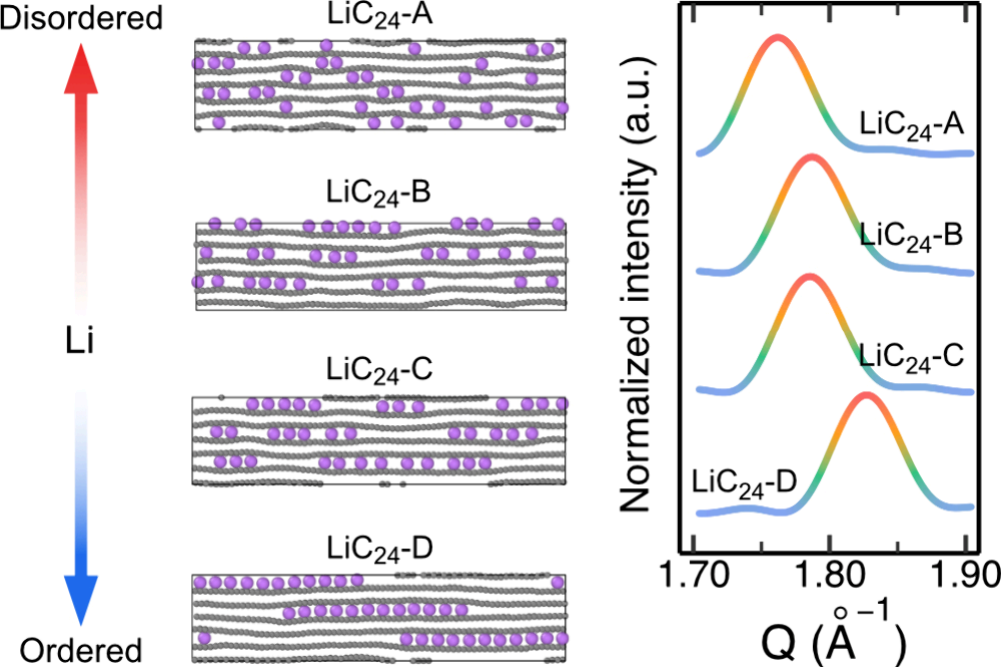
RE-MD simulations of Li-ordering in LiC_24_. Simulated LiC_24_ structures (left) and the corresponding (002) peak positions (right). Despite having the same Li concentration, the different arrangements lead to significant peak shifts.

The simulated XRD patterns for LiC_24_-A to LiC_24_-D reveal that the position of the (002) reflection is determined not only by Li ion concentration but also by Li ion distribution. The increasing structural order is evident from the sharpening of both (002) and (101) reflections (Figure S6). This indicates that the peak shifts observed during relaxation could be caused by structural rearrangements. Even though the only differences between the LiC_24_-C and LiC_24_-D structures are the size and density of the Li ion islands, the (002) reflection position in the *Q*-space still shifts by 0.05 Å^–1^.

The high-time resolution of the operando measurements allowed us to carry out a detailed assessment of the kinetics of the relaxation process of graphite. We extracted the changes in the (002) reflections of LiC_18_/LiC_30_ and (101) reflection of LiC_30_ from a surface peak fit of the relaxation regions of the operando XRD dataset (full details related to the fitting are given in the Supporting Information). The declining intensity of the LiC_18_ (002) peak was used as an indicator of progress of the LiC_18_–LiC_30_ reverse phase change, while the positions of the LiC_30_ (002) and (101) peaks are used to track the subsequent changes during the relaxation of LiC_30_ (Figure S5). Plotting ln(−ln(1 – α)) vs ln(*t* – *t*_0_) in the logarithmic form of the Kolmogorov–Johnson–Mehl–Avrami (KJMA) theory (eq 1) allows us to extract the kinetic information:
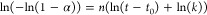
1where α is the normalized phase-representative parameter for the process being analyzed (see Figure S5 and Supplementary Note 1), *k* is the rate constant of reaction, *t*_0_ is the induction time, (*t* – *t*_0_) is the time elapsed from the beginning of the relaxation process, and *n* is the Avrami exponent. This method (i.e., the Sharp–Hancock plot) allows for determining the rate and dimensionality of the process being studied (Figure 4), with extracted Avrami exponents *n* and rate constants *k* (Table S2).^45,46^ By identifying regions with different linear slopes in the Sharp–Hancock plots (Figure 4), we can divide the relaxation processes in graphite into three different relaxation stages, which can be rationalized with the aid of the RE-MD simulation described above (Figure 3).

**Figure 4:**
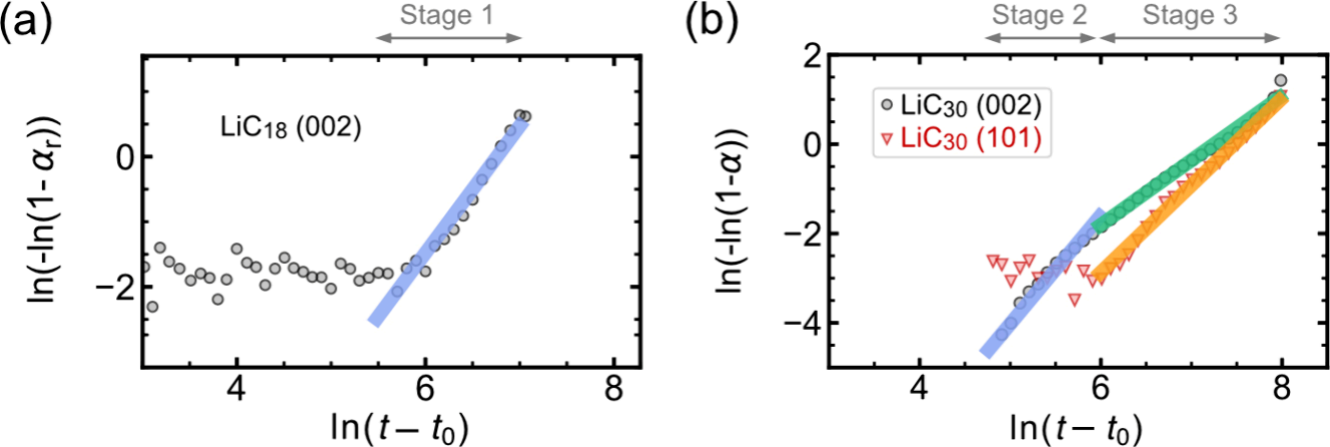
Kinetics of relaxation. The Sharp–Hancock plot and fitted Avrami slope derived from (a) the intensity changes in the (002) reflection of LiC_18_ in the operando XRD data during the reversal of the LiC_18_ to LiC_30_ transition, and (b) the positional changes of the (002) and (101) reflections during the relaxation of LiC_30_.

**Relaxation stage 1** (Figure 4a) is a reversal of the first lithiation phase, i.e., the LiC_18_ phase at the nonequilibrium state relaxes back to LiC_30_, traced by the intensity changes of the disappearing LiC_18_ (002) peak between 1.42 h and 1.75 h from the start of the experiment (Figure 1). This is a fast process with an Avrami exponent and rate constant of 1.95 ± 0.048 and 0.0019 s^–1^, respectively. The LiC_30_ (002) peak position is almost constant as the LiC_18_ (002) peak disappears. There are no changes in the position of the (101) reflection. Therefore, we believe that relaxation stage 1 consists of the Li clusters of the LiC_18_ structure break up and return to an arrangement more like LiC_30_. As the LiC_30_ arrangement re-established itself the spaces between the Li ions in the galleries are small and so the gallery height does not change.^42^ Notably, the AA stacking of graphene sheets remains during the reverse phase transition, as evidenced by the lack of observable changes in (101) reflection position.

**Relaxation stage 2** (blue line in Figure 4b) is a reorganization of Li ions within the LiC_30_ phase, described by the shift in the position of (002) peak between 1.75 and 1.86 h (Figure 1). The calculated structures LiC_24_-B and LiC_24_-C (Figure 3) illustrate the underlying mechanism of Li reorganization at this stage. The observed shift in the (002) reflection is directly related to reduction of the average gallery height. Like relaxation stage 1, this is a fast process with a related Avrami exponent of 2.43 ± 0.097 and a rate constant of 0.0019 s^–1^. The position of the (101) peak (related to the graphite layer stacking and gallery height; see Figure 1f) does not change during this stage of relaxation. Our models suggest that small Li clusters relatively short distances apart hold the galleries open and retain the AA graphene sheet stacking encountered in fully lithiated galleries.^42^ Some slightly larger empty regions in the galleries, scattered throughout the crystallites, begin to contract to graphite-like heights and revert to AB stacking but do not yet have a significant influence on the diffraction pattern.

**Relaxation stage 3** (green and orange lines in Figure 4b) is the densification of Li-clusters into larger islands, indicated by the (002) and (101) peak position changes between 1.86 and 2.55 h (Figure 1). We identified Avrami exponents of 1.45 ± 0.003 and 1.95 ± 0.007 for the shifts of the (002) and (101) peaks, respectively, and rate constants of 0.0014 s^–1^ and 0.0010 s^–1^. As a result of the formation of Li islands, large empty regions are left in the galleries. In these, the gallery height reverts to a graphite-like value,^42^ and the graphene sheets move laterally to re-establish the AB stacking pattern of the parent material. This rate constant indicates that the lateral slip relaxes more slowly than the gallery height, which can be explained by the dimensionality of Li-island ordering and the occasional rotation or displacement of graphene sheets in turbostratic stacking domains. Close inspection of the operando XRD patterns of graphite (Figure 1 and Figure S1) reveals that the (101) reflection position does not start to drift until the (002) reflection has already shifted significantly. This indicates that rather than continuing to form more LiC_30_ or change the degree of lithiation (i.e., forming LiC_18_), the relaxation process is instead driven by Li rearrangement and changes in the gallery stacking mode to create the more thermodynamically favorable combination of Li islands and larger empty areas in which the graphite gallery height and AB- stacking sequence can be re-established.

The relaxation behavior of graphite shows that the nonequilibrium structures LiC_18_ and LiC_30_ rearrange to more thermodynamically favorable structures upon removal of the external potential. This relaxation happens because of a competition between two forces: (1) the attractive van der Waals interactions between graphite interlayers in AB stacking mode keep the planes free of Li ions, and (2) the repulsive interactions between Li ions keep them as far apart as possible within the same gallery. The conventional understanding of LiC_*x*_ phases, where crystal structures are built up of (essentially) full galleries with AA stacking and LiC_6_ gallery height and empty galleries with AB stacking and graphite gallery height, is not sufficient to explain the kinetics we observe, as AA and AB stacking would always be present. However, the intermediate structures modeled by RE-MD offer a reasonable explanation, driven by the tendency of Li ions to form clusters and, eventually, larger Li islands and empty regions that create local domains of AA and AB stacking (Figure 5a).

**Figure 5:**
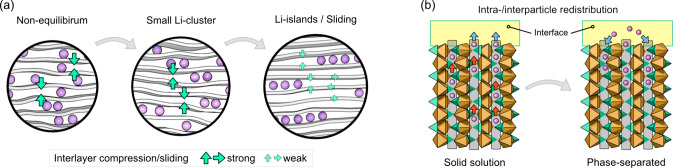
Schematic illustration of the relaxation mechanism. The relaxation mechanism of LiC_30_ is stepwise and driven by local Li rearrangement, forming Li clusters and Li islands.

LFP exhibits a much slower reorganization of Li ions than graphite. Eight hours were needed to stabilize the changes in the XRD peak intensity and position (Figure 2). We think that this slow reorganization is an attribute of the chemical nature of the channels in the materials. Furthermore, the curvatures of the extracted intensity changes of LFP and FP (Figure 2b) could indicate that there are multiple relaxation processes observed for this electrode happening at different time scales, similarly to the description by Li et al.^40^ The relaxation process of partially lithiated LFP/FP particles can be understood, like graphite, in terms of the redistribution of Li ions (Figure 5b). The stabilization of pure LFP and FP phases during relaxation can be compared to the empty and filled gallery regions in graphite, where Li ions concentrate either in parts of the larger particles or in phase-segregated small LFP domains (intraparticle and interparticle redistribution). A partially (de)lithiated LFP electrode at rest will relax to the equilibrium two-phase state of lithiated and delithiated LFP.^23^

In summary, we have used operando XRD to directly observe the structural changes during relaxation of two common battery materials, graphite and LFP, after the interruption of cycling. Using RE-MD models and kinetic analysis, we have shown that the relaxation of both is driven by clustering of the Li ions to achieve the most thermodynamically stable arrangement. These results are significant for any kind of analysis or testing of battery materials that involves interruption of the electrochemistry: Understanding the kinetics of relaxation and the underlying structural changes on the atomic level should be a basic guideline for interpreting structural data collected after interruptions of cycling, parametrization of GITT, and design of battery cycling protocols. The intricacies of the relaxation process revealed in this work may also help us better understand how materials behave during the phase transitions that occur during cycling. We believe that, even though the relaxation mechanism is material-specific, related processes will occur for any active material in metal-ion batteries. The time scales and structural end points will depend on the point of interruption and the structures that are present at that time.

## Supporting Information Available

Experimental details for the fabrication and components of the operando cell, operando measurements, data processing and analysis and details regarding RE-MD (PDF)
