# Supplementary material for: Stepwise Structural Relaxation in Battery Active Materials
Source: ACS Mater Lett. 2024 Dec 23;7(1):343–9. doi: 10.1021/acsmaterialslett.4c02058 (PMC11707793; doi:10.1021/acsmaterialslett.4c02058)
Supplement: Supplementary file 1 — tz4c02058_si_001.pdf [file tz4c02058_si_001.pdf]

Supporting Information for

Stepwise Structural Relaxation in Battery Active Materials

Amalie Skurtveit[1], Erlend Tiberg North[1], Heesoo Park[1], Dmitry Chernyshov[2], David S. Wragg[1,3*], Alexey Y. Koposov[1,3*]*

[1]Centre for Materials Science and Nanotechnology, Department of Chemistry, University of Oslo, PO Box 1033, Blindern, 0315 Norway

[2] Swiss-Norwegian Beamlines, European Synchrotron Facility, 71 Avenue des Martyrs, 38000 Grenoble, France

[3] Department of Battery Technology, Institute for Energy Technology, Instituttveien 18, 2007 Kjeller, Norway

* Corresponding authors at: amalie.skurtveit@kjemi.uio.no, david.wragg@ife.no, alexey.koposov@kjemi.uio.no

Table of Contents

Experimental section 3

Electrode preparation and cell assembly....3

Operando XRD 3

Table S1: Experimental conditions during *operando* XRD measurements 5

Molecular dynamics (MD) simulation 6

Parametric peak fits and relaxation kinetics....6

Morphology 7

Fig. S1: ***Operando*** **XRD characterization of (101) reflection of graphite during relaxation.** 9

Fig. S2: ***Operando*** **XRD characterization of graphite from an LG JP3 pouch cell cycled at C/20.** 10

Fig. S3: ***Operando*** **XRD characterization of relaxation of the (002) reflection of** LiC_x**.** 12

Fig. S4: ***Operando*** **XRD characterization of relaxation of the (002) reflection of** LiC_x**.** 13

Table S2: Avrami exponents, n, and rate constants, k....14

Fig. S5: α values used to follow different kinetic processes.....15

Fig. S6: Full width at half-maximum (FWHM) obtained from peak fitting of reflections during the relaxation of LiC_{30}**.**....15

Supplementary references....16

Experimental section

Electrode preparation and cell assembly

The graphite-based electrodes were removed from an uncycled LG JP3 pouch cell. One side of the electrode was cleaned to remove the active material, while the other side remained untouched, before punching into Ø15 mm disks for use in CR2032 coin cells. The LFP-based electrodes were purchased from CustomCells® and had an areal capacity of 1.0 mA cm^{-2}. The electrode was placed into an *operando* cell, similar to the one described by Drozhzhin *et al.*, equipped with glassy carbon windows.[1] All cells were assembled in an Ar-filled glovebox (MBraun LabStar) with H_2O and O_2 levels below 0.5 ppm. Li foil was used as the counter electrode, while glass fiber (Whatman GFP/C) was used as separator, soaked in 80 µL of 1.2 M $LiPF_6$ in ethylene carbonate (EC):ethyl methyl carbonate (EMC) (3:7 vol.) + 2 wt.% vinylene carbonate (VC) and 10 wt.% fluoroethylene carbonate (FEC) electrolyte (Solvionic). The cells were tested using galvanostatic cycling (GC) by using a Bat-Small battery cycler (Astrol) between 0.01 V and 2.0 V *vs* Li/Li^+ for graphite, and between 2.0 V and 4.2 V *vs* Li/Li^+ for the LFP-based electrode.

Operando XRD

The *operando* XRD measurements were carried out at the BM01 beamline at the Swiss-Norwegian Beamlines (SNBL), ESRF in Grenoble, France. BM01 is equipped with a Dectris Pilatus 2M detector and monochromatic radiation with a wavelength of 0.68316 Å was used for the relaxation studies of graphite, 0.72189 Å was used for the C/20-study of graphite, and 0.68922 Å was used for LFP. The 2D diffractograms were azimuthally integrated to give 1D

diffraction patterns using Bubble.[2] The potentiostat was placed outside the experimental hutch and the connection to the cell was directed through a chicane. To investigate the relaxation process, the connection from the potentiostat to the cell was physically unplugged to exclude experimental discrepancies due to the inaccuracy of the electrochemical tester, while the diffraction data acquisition continued. The count time for LFP was kept constant at 10 seconds per diffractogram throughout the whole experiment. For graphite, the count time was 10 seconds until the start of the relaxation, when we switched to a count time of 1 second. For better powder averaging, the cell was rotated with a 10° rotation about the φ axis during each exposure. Reversal of this rotation before the next exposure took approximately 0.24 seconds, giving the experimental time resolution.

The graphite-based electrodes used for *operando* XRD characterization, including relaxation (Fig.1, Supplementary Fig. S1, S3, S4) and the electrochemical behavior study (Supplementary Fig. S2), are extracted from the same uncycled LG JP3 pouch cell. For the graphite-based electrode, a total of four cells were used to conduct our studies; three were used for investigating the relaxation mechanism in graphite, and one for comparison of the electrochemical behavior, (cycled at C/20 for the first (de)lithiation, then C/4). Supplementary Table S1 summarizes the experimental conditions for each cell.

Table S1: Experimental conditions during *operando* XRD measurements

Measurement	Electrode	Wavelength (Å)	C-rate[1]	Figures
Relaxation LiC_{30}	Ø15 mm from LG JP3 pouch cell	0.68316	C/6[2]<br>C/2[3]	Fig. 1,<br>Fig. S1
Relaxation LiC_{18}		0.68316	C/6	Fig. S4
Relaxation LiC_{30}		0.68316	C/3	Fig. S5
C/20-cycling		0.72189	C/20[4]<br>C/4	Fig. S2
Relaxation LFP	Ø15 mm from CustomCells® areal capacity 1.0 mA cm^{-2}	0.68922	C/4	Fig. S2

[1]Practical C-rates, [2]C-rate for the first lithiation, [3]C-rate for the first delithiation, [4]C-rate for the first cycle

Molecular dynamics (MD) simulation

Molecular dynamics (MD) simulations used the ReaxFF reactive force field in the isothermal-isobaric (NPT) ensemble with periodic boundary conditions. We adopted the set of reactive force field parameters that Raju *et al.* developed to describe Li-interactions in carbon-based materials for the storage of electrical energy based on van der Waals corrected density functional theory,[3] employing replica exchange MD (RE-MD) method to accelerate Li rearrangements during the simulations.[4] MD simulations were carried out using the parallel reactive molecular dynamics module in the LAMMPS package.[5-8] As the method intrinsically involves simulations at several temperatures, we present the properties replica corresponding to the room temperature (302.22 K) as the most representative for supporting experimental data.

Surface peak fits and relaxation kinetics

Surface peak fits were performed on the relaxation region of the *operando* XRD datasets in TOPAS v.6.[9, 10] We used 3rd order Chebyshev polynomial to model the background contributions and assumed Gaussian peak shapes. From the surface peak fits, we extracted the intensity and peak position of the following reflections: (002) for LiC_{18} and LiC_{30}, and (101) for LiC_{30}. The benefit of the surface method is that parameters, such as zero error (which is

unlikely to change during the course of the experiment), can be refined as a single variable against the entire surface of data.

To assess the relaxation kinetics, we applied the linearized form of the Kolmogorov-Johnson-Mehl-Avrami (KJMA) theory (eq.1 in Main) by normalizing the change in the intensity or peak position for the extracted reflections.[5-8] This is referred to as the phase-representative parameter, α. The α-parameter for the different analyzed processes are as follows:

- α-parameter for LiC_{18} is the normalized changes in the intensities of the (002) reflection, shown in Fig. S5a. Q-range: 1.84 to 1.85 $Å^{-1}$.
- α-parameter for LiC_{30} is the normalized changes in the peak position of the (002) and (101) reflection, shown in Fig. S5b. Q-range (002) reflection: 1.86 to 1.87, and Q-range for (101) reflection: 3.093 to 3.096 $Å^{-1}$.

Normalization of the α-parameter was done by min-max feature scaling, a common approach for comparisons.

Linearizing the KJMA relationship yields a straight line of slope n and intercept $n\ln(k) - \ln(e)$, where we extracted the Avrami exponents, n, and rate constants, k, shown in Fig. 4 in Main.

Morphology

The morphology of the electrodes was studied with a high-resolution Hiatchi SU8230 cold-field emission scanning electron microscope (SEM) using an acceleration voltage of 10 kV.

Preparation of the samples was carried out in an Ar-filled glovebox (MBraun LABmaster) with O_2 and H_2O levels below 0.1 ppm to minimize structural changes to the dried electrodes. However, the samples were shortly exposed to ambient conditions right before transferring them to the SEM, but this minor air exposure should not affect the morphology of the samples. The surface and cross-sections of the electrodes were obtained by cutting out a small piece of the electrode and placing it on carbon tape to prevent sample movement.

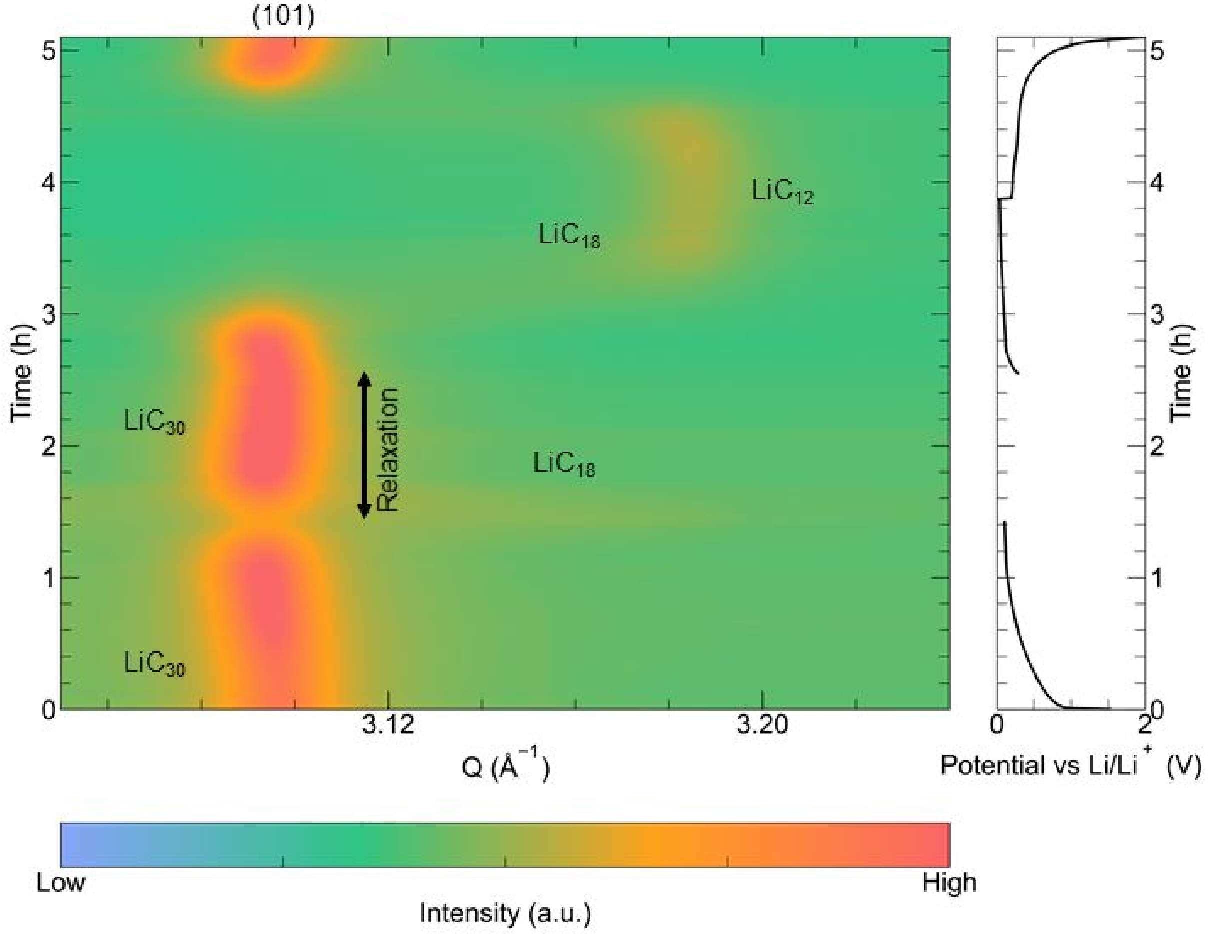

Fig. S1: ***Operando*** **XRD characterization of (101) reflection of graphite during relaxation.** The evolution of (101) reflection of the graphite-based electrode as a function of lithiation and relaxation, corresponds to the right panel in Fig. 1. Note that the intensity of the peaks is scaled to make small details in the reflections more pronounced compared to Fig.1 in Main text.

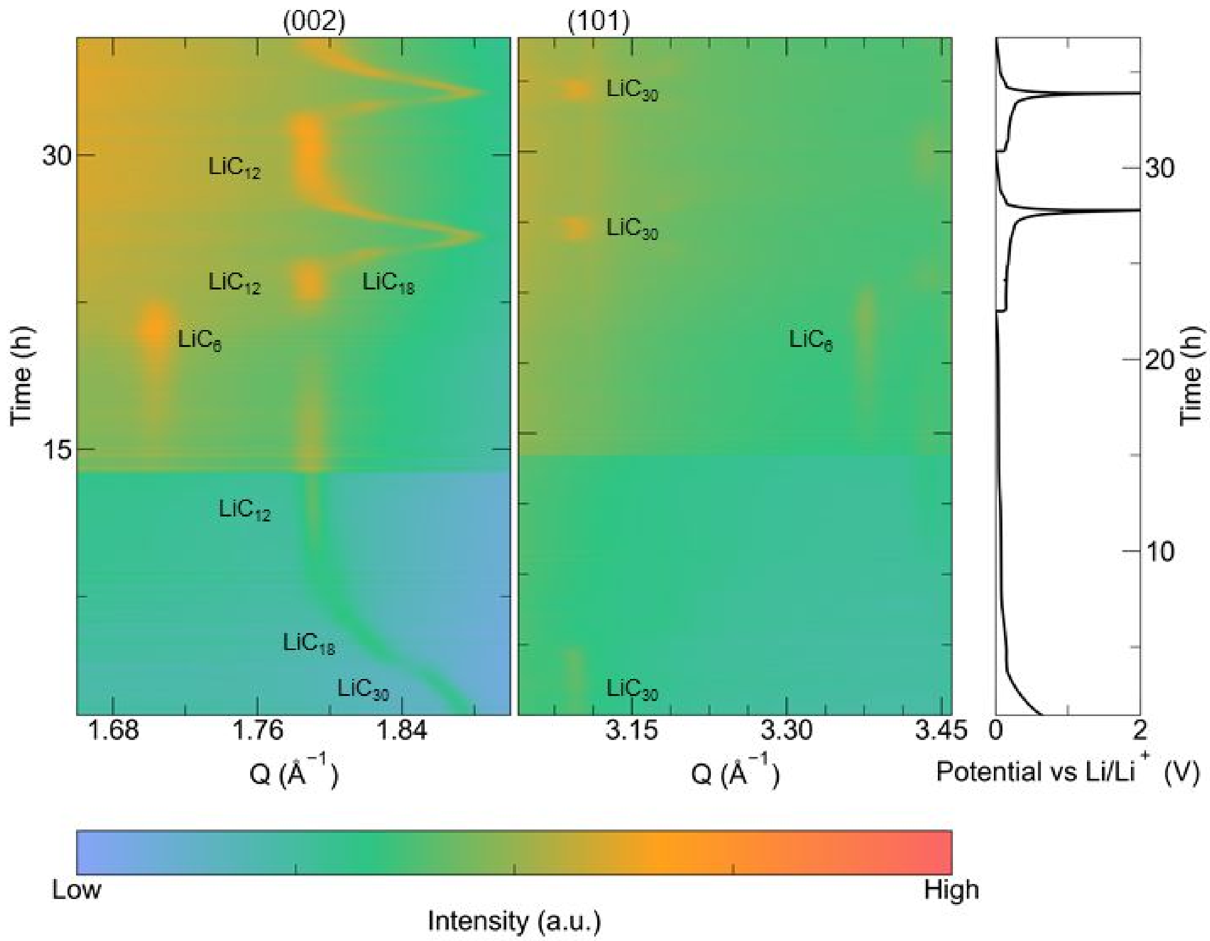

Fig. S2: ***Operando*** **XRD characterization of graphite from an LG JP3 pouch cell cycled at C/20.** The evolution of the (002) and (101) reflections of graphite as a function of state of (de)lithiation. The first cycle was collected using a C-rate of C/20, while the 2nd and 3rd (de)lithiation was collected using a C-rate of C/4. At the end of the first lithiation, LiC_6 is formed as expected when cycling under these conditions. In the 2nd and 3rd lithiations (C/4), only LiC_{12} is formed.

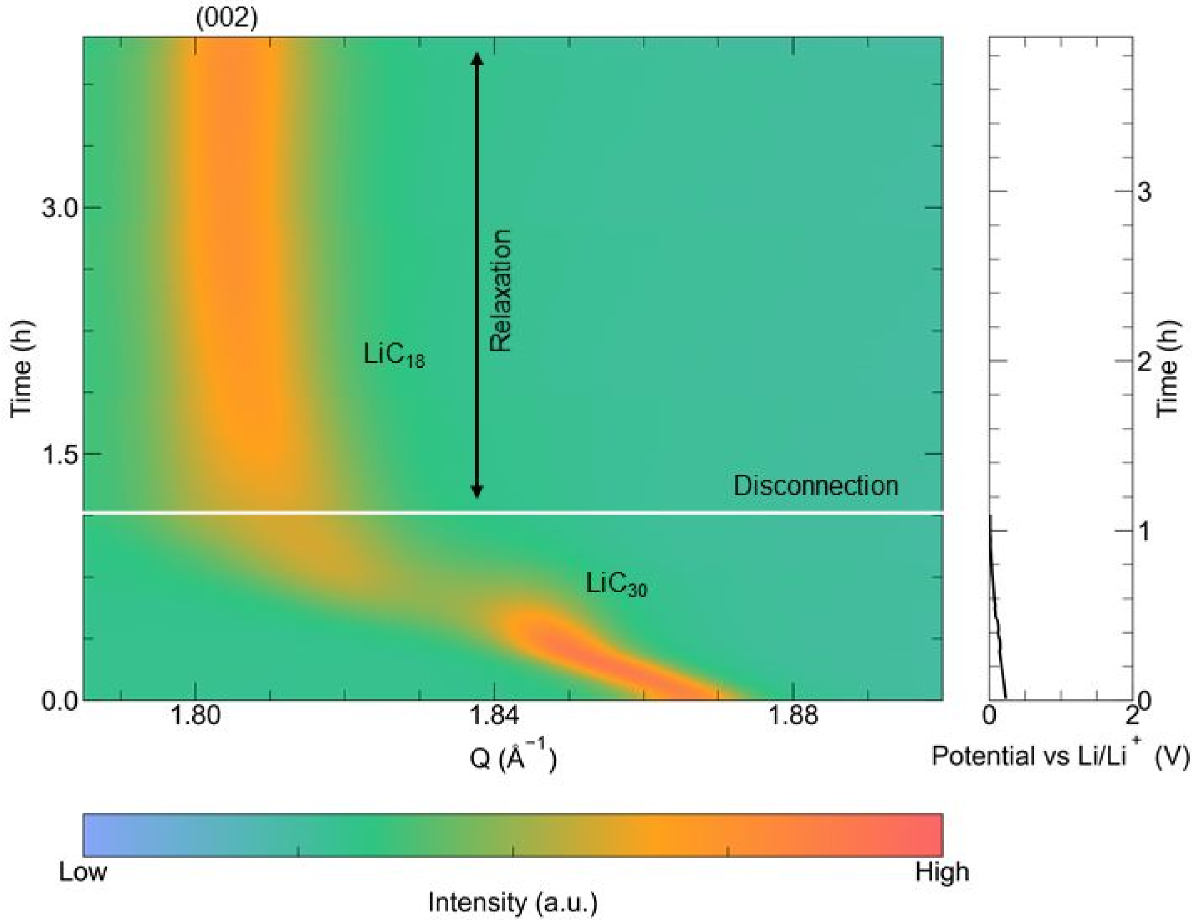

Fig. S3: ***Operando*** **XRD characterization of relaxation of the (002) reflection of LiC_x.** Tracking the (002) reflection of graphite from an LG JP3 pouch cell during lithiation and relaxation. Relaxation was induced after the LiC_{30} to LiC_{18} phase transition (marked with a white line) at 0.012 V *vs* Li/Li$^+$, where the (002) reflection suggests that LiC_{18} relaxes by reorganization of Li-ions and graphite sheets towards an equilibrium state. The shift of the peak to lower Q-values in the relaxation can be explained by the presence of interstitial Li-ions in domains that have an excess of Li-ions. In these domains, the repulsive Li—Li interaction causes Li-ions to repel each other, which in turn promotes the diffusion of Li-ions and widens the gallery heights

at the adjacent Li-free sites. This structural reorganization contrasts the behavior observed at a lower lithium content (i.e., LiC_{30} shown in Fig. 1).

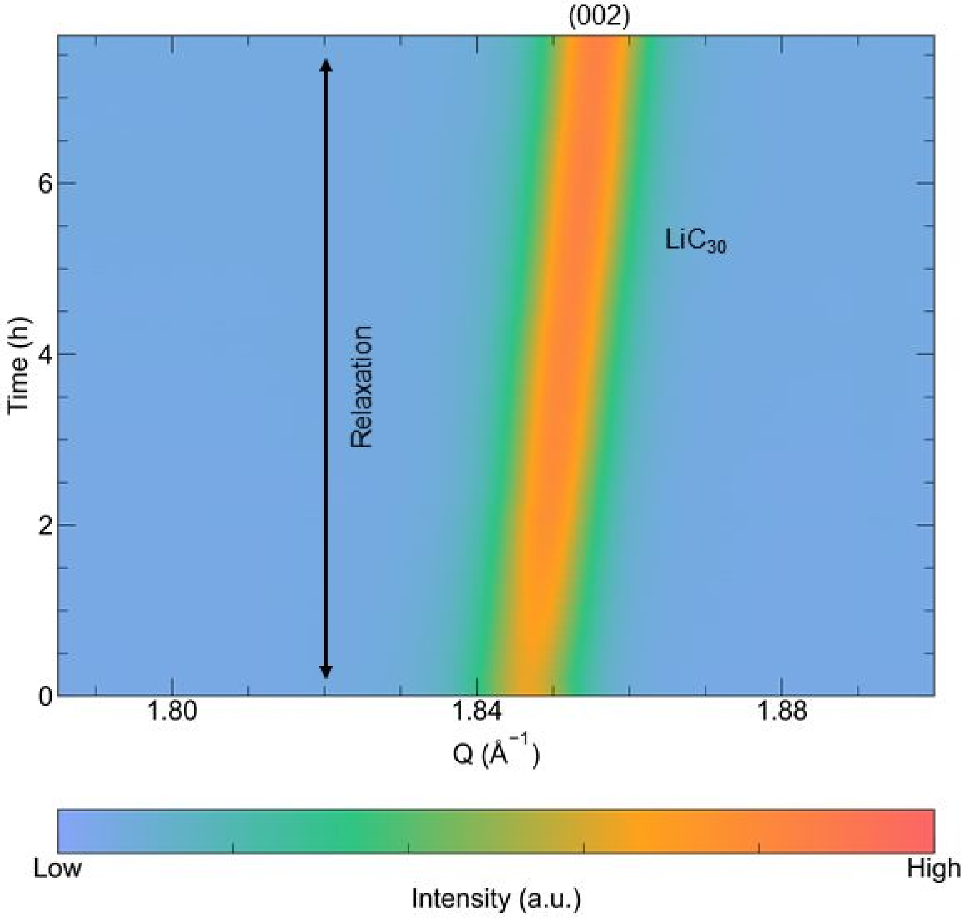

Fig. S4: *Operando* XRD characterization of relaxation of the (002) reflection of LiC_x. Relaxation of LiC_{30} after interruption of electrochemistry at 0.078 V. Structural relaxation in LiC_{30} is driven by local Li-redistribution within the galleries towards the most thermodynamically favorable configuration. The relaxation progresses identically to what is described in the Main text, see Fig. 1.

Table S2: Avrami exponents, n, and rate constants, k, derived from the linear fits of the Sharp-Hancock plots (Fig. 4 in Main) at a given time interval.

**(Reflection) material stage**	**Start time t_0 (s)**	**End time, t (s)**	**Time interval (h)**	**Avrami exponent, n**	**Rate constant, k (s^{-1})**
(002) LiC_{18} Stage 1	60	1180	1.44–1.75	1.95±0.048	0.0019±9.7×10^{-5}
(002) LiC_{30} Stage 2	1180	1580	1.75–1.86	2.43±0.097	0.0019±2.4 ×10^{-4}
Stage 3	1580	4080	1.86–2.55	1.45±0.003	0.0014±5.7×10^{-7}
(101) LiC_{30} Stage 3	1580	4080	1.86–2.55	1.95±0.007	0.0010±1.4×10^{-6}

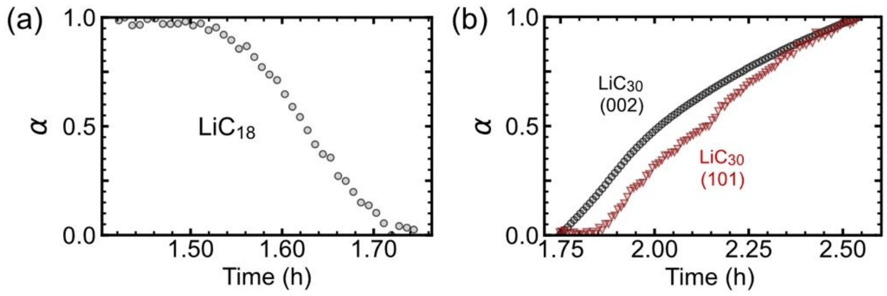

Fig. S5: α values used to follow different kinetic processes. (a) Normalized intensities of the (002) reflection of LiC_{18} during the reversal of the LiC_{18} to LiC_{30} transition, (b) normalized peak positions of the (002) and (101) reflections of LiC_{30}.

Supplementary Note 1: Note that the changes in LiC_{18} in Figure 4 in Main are represented as a growth process, rather than a disappearance by reversing the α-parameter (i.e., $\alpha_r = 1 - \alpha$).

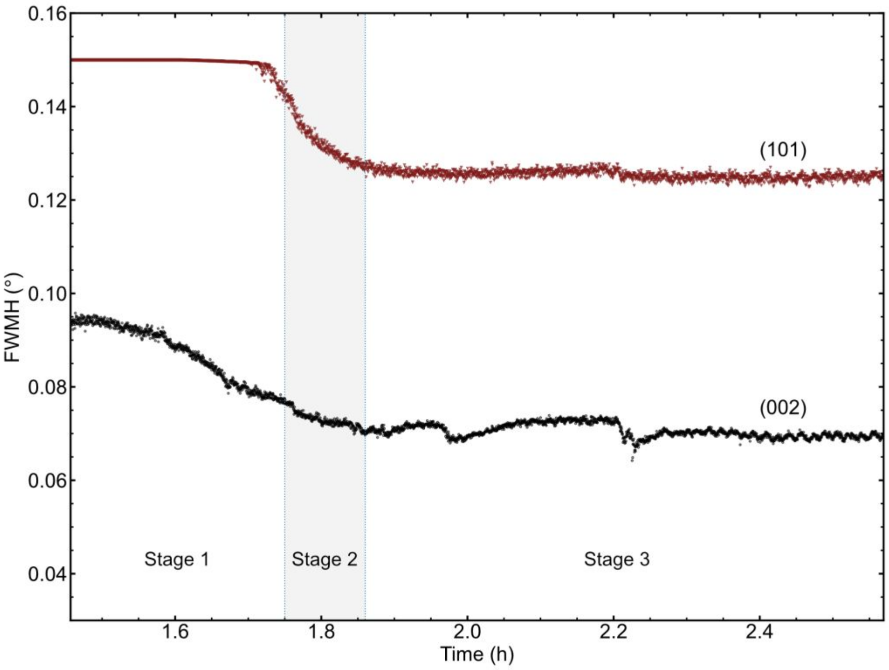

Fig. S6: Full width at half-maximum (FWHM) obtained from peak fitting of reflections during the relaxation of LiC_{30}.

Supplementary references

(1) Drozhzhin, O. A.; Tereshchenko, I. V.; Emerich, H.; Antipov, E. V.; Abakumov, A. M.; Chernyshov, D. An electrochemical cell with sapphire windows for operando synchrotron X-ray powder diffraction and spectroscopy studies of high-power and high-voltage electrodes for metal-ion batteries. *Journal of Synchrotron Radiation* **2018**, *25* (2), 468-472. DOI: 10.1107/S1600577517017489.

(2) Dyadkin, V.; Pattison, P.; Dmitriev, V.; Chernyshov, D. A new multipurpose diffractometer PILATUS@SNBL. *Journal of Synchrotron Radiation* **2016**, *23* (3), 825-829. DOI: 10.1107/S1600577516002411.

(3) Raju, M.; Ganesh, P.; Kent, P. R. C.; van Duin, A. C. T. Reactive Force Field Study of Li/C Systems for Electrical Energy Storage. *Journal of Chemical Theory and Computation* **2015**, *11* (5), 2156-2166. DOI: 10.1021/ct501027v.

(4) Park, H.; Wragg, D. S.; Koposov, A. Y. Replica exchange molecular dynamics for Li-intercalation in graphite: a new solution for an old problem. *Chemical Science* **2024**, *15* (8), 2745-2754, 10.1039/D3SC06107H. DOI: 10.1039/D3SC06107H.

(5) Thompson, A. P.; Aktulga, H. M.; Berger, R.; Bolintineanu, D. S.; Brown, W. M.; Crozier, P. S.; in 't Veld, P. J.; Kohlmeyer, A.; Moore, S. G.; Nguyen, T. D.; et al. LAMMPS - a flexible simulation tool for particle-based materials modeling at the atomic, meso, and continuum scales. *Computer Physics Communications* **2022**, *271*, 108171. DOI: 10.1016/j.cpc.2021.108171.

(6) Aktulga, H. M.; Fogarty, J. C.; Pandit, S. A.; Grama, A. Y. Parallel reactive molecular dynamics: Numerical methods and algorithmic techniques. *Parallel Computing* **2012**, *38* (4), 245-259. DOI: 10.1016/j.parco.2011.08.005.

(7) Okabe, T.; Kawata, M.; Okamoto, Y.; Mikami, M. Replica-exchange Monte Carlo method for the isobaric–isothermal ensemble. *Chemical Physics Letters* **2001**, *335* (5), 435-439. DOI: 10.1016/S0009-2614(01)00055-0.

(8) Mori, Y.; Okamoto, Y. Generalized-Ensemble Algorithms for the Isobaric–Isothermal Ensemble. *Journal of the Physical Society of Japan* **2010**, *79* (7), 074003. DOI: 10.1143/JPSJ.79.074003 (acccessed 2023/09/18).

(9) Coelho, A. TOPAS and TOPAS-Academic: an optimization program integrating computer algebra and crystallographic objects written in C++. *Journal of Applied Crystallography* **2018**, *51* (1), 210-218. DOI: 10.1107/S1600576718000183.

(10) Stinton, G. W.; Evans, J. S. O. Parametric Rietveld refinement. *Journal of Applied Crystallography* **2007**, *40* (1), 87-95.
